# Relationship between depression, anxiety, and perceived burdensomeness: the mediating role of emotional regulation among Egyptian older adults

**DOI:** 10.3389/fpubh.2026.1780055

**Published:** 2026-04-14

**Authors:** Ibrahim Alasqah, Ahmed Abdelwahab Ibrahim El-Sayed, Ahmed Abdellah Othman, Mohamed Hussein Ramadan Atta, Noha Gamal El-Sayed Ghoniem, Shaimaa Mohamed Amin

**Affiliations:** 1 Department of Community, Psychiatric, and Mental Health Nursing, College of Nursing, Qassim University, Buraydah, Saudi Arabia; 2 Nursing Administration Department, Faculty of Nursing, Alexandria University, Alexandria, Egypt; 3 Nursing Administration Department, Faculty of Nursing, Sohag University, Sohag, Egypt; 4 Nursing Department, College of Applied Medical Sciences, Prince Sattam Bin Abdulaziz University, Wadi Addawasir, Saudi Arabia; 5 Psychiatric and Mental-Health Nursing Department, Faculty of Nursing, Alexandria University, Alexandria, Egypt; 6 Department of Gerontological Nursing, Faculty of Nursing, Zagazig University, Zagazig, Egypt

**Keywords:** anxiety, depression, emotional regulation, empowerment, older adults, perceived burdensomeness

## Abstract

**Objective:**

To examine whether emotion regulation moderates the relationship between depression, anxiety, and perceived burdensomeness among community-dwelling older adults with attention to its relevance for public health.

**Methods:**

A cross-sectional study was conducted among 300 adults aged ≥60 years attending outpatient clinics in Egypt. Validated instruments measured emotion regulation, depression, anxiety, and perceived burdensomeness. Pearson’s correlation and multivariable regression analyses were applied to examine relationships among variables and evaluate moderation effects.

**Results:**

Perceived burdensomeness was positively associated with depression and anxiety (*r* = 0.765, *p* < 0.001) and negatively with emotion regulation (*r* = −0.676, *p* < 0.001). Emotion regulation predicted lower burdensomeness (*β* = −0.54, *p* < 0.001) and reduced depression, anxiety, and stress (*β* = −0.43, *p* < 0.001). Better regulation skills attenuate the negative impact of mental health symptoms on burdensomeness.

**Discussion:**

Emotion regulation acts as a buffer, mitigating distress and perceived burdensomeness in later life. Integrating emotion regulation training into geriatric programs may enhance resilience and reduce suicide risk in older adults. These results highlight the need to integrate emotion regulation strategies into public health initiatives, especially within primary care and community services that serve older populations.

## 1 Introduction

Aging brings increased risks of mental health issues such as depression and anxiety. Additionally, physical conditions like chronic pain, frailty, mobility impairments, and dementia frequently co-occur, further impacting mental health and increasing the need for long-term care (1). Approximately 14% of people aged 60 and above experience a mental disorder, accounting for 10.6% of the total years lived with disability for this age group.

Depression and anxiety are the most common conditions. Alarmingly, over one-quarter (27.2%) of global suicide deaths occur in this age group (1). Unfortunately, these disorders are often underdiagnosed and undertreated due to stigma and lack of awareness. Mental well-being in later life is influenced by both past life experiences and current stressors associated with aging. Psychological distress can result from reduced capacity, bereavement, retirement, and income decline. Ageism and societal undervaluing of older adults further threaten mental health (2).

Emotion regulation is a vital psychological skill that enables individuals to manage the emotional fluctuations of everyday life. The capacity to identify, comprehend, and flexibly regulate emotions is essential for psychological health, overall well-being, and effective functioning throughout all stages of life (3). Nevertheless, many people encounter difficulties with regulating their emotions, which can result in various personal and social issues. These emotion regulation deficits are commonly observed in psychological disorders such as anxiety, depression, and substance use disorders (4).

Emotion regulation plays a pivotal role in maintaining mental health across the lifespan. Older adults often exhibit more adaptive emotion regulation strategies, such as cognitive reappraisal, which contribute to greater emotional stability and well-being (3–5). This emotional expertise is associated with reduced symptoms of anxiety and depression, particularly as individuals age and prioritize emotionally meaningful experiences over novelty-seeking behaviors (6, 7). Christopher and Facal (8) emphasize that older adults, despite stereotypical associations with decline, often demonstrate emotional resilience, which supports their overall mental health.

## 2 Background

While much of the attention and research on suicide has traditionally centered around adolescents and young adults, suicide among older adults is a significant issue both in the United States and globally. In 2013, the overall suicide rate in the U.S. was 13 per 100,000 individuals, but among those aged 65 and older, the rate rose to 16.1 per 100,000. This rate further increased with age, reaching 18.6 per 100,000 among individuals aged 85 and above (9). Furthermore, evidence indicates that suicidal behavior in older adults is far more likely to result in death compared to younger populations (10). These alarming statistics highlight the critical need to understand the risk factors associated with suicide in later life.

One influential and relatively recent explanation is Thomas Joiner Jr.’s interpersonal theory of suicide (11, 12), which has been widely studied across various populations. This theory, also known as the interpersonal-psychological theory, suggests that the desire for suicide arises from two core factors: thwarted belongingness and perceived burdensomeness. A third factor, the acquired capability for lethal self-harm, contributes to the likelihood of suicide attempts being fatal. Thwarted belongingness refers to feelings of isolation and lack of meaningful social connections, while perceived burdensomeness involves the belief that one is a burden on others, sometimes to the extent of believing that others would be better off if they were dead (11). According to Van Orden et al. (12), perceived burdensomeness includes two key elements: viewing oneself as a liability to loved ones and harboring intense self-hatred.

Research has shown a significant link between perceived burdensomeness and suicidal thoughts and behaviors, even after accounting for other variables such as depression and hopelessness. For example, among adult outpatients, higher levels of perceived burdensomeness were strongly associated with suicidal ideation and attempts (13). Similarly, a study examining the full interpersonal theory in college students found that both perceived burdensomeness and thwarted belongingness were independently related to suicidal ideation, with the combination of both leading to even stronger suicidal thoughts (14). This correlation has also been observed in community-based studies (15, 16). A review of clinical research further supports a clear connection between perceived burdensomeness and suicidal behaviors, suggesting that this perception may mediate or moderate the relationship between known risk factors, such as depression, and suicide-related outcomes (17).

Perceived burdensomeness, the belief that one is a burden to others, has been consistently linked to negative psychological outcomes, including lower self-worth, depression, and suicidal ideation (18). Amin et al. (19) found a strong negative correlation between perceived burdensomeness and psychological well-being in older adults with chronic illnesses, where higher burdensomeness predicted lower well-being. Furthermore, Gager et al. (20) identified that perceived burdensomeness contributes to feelings of hopelessness and emotional distress, making it a critical variable in understanding older adults’ mental health trajectories.

As people grow older, their emotion regulation capabilities often become more refined and effective. Older adults typically report fewer negative emotions and display better emotional well-being than younger individuals (5, 6). This enhanced emotional regulation may stem from several factors, including greater emotional knowledge, improved cognitive control, and motivational changes that prioritize emotionally meaningful goals (4, 7). However, aging also brings unique challenges, such as cognitive decline, worsening physical health, and bereavement, which can complicate emotional regulation.

According to socioemotional selectivity theory, aging shifts individuals’ focus from seeking new information and experiences to concentrating on emotionally rewarding goals, like sustaining close relationships and finding life’s meaning (6). This motivational shift contributes to the way emotion regulation strategies evolve with age. Older adults often exhibit superior emotion regulation, particularly in their use of cognitive reappraisal, an approach that involves reframing the meaning of a situation to alter its emotional impact (7). They also tend to report greater emotional positivity, a pattern known as the “positivity effect.” At the same time, aging may be associated with a preference for more passive regulation strategies, such as avoidance or emotional disengagement, which could be attributed to reduced cognitive resources, lower energy levels, or diminished motivation to exert regulatory effort (3).

Although existing literature highlights the generally enhanced emotion regulation abilities of older adults and their positive impact on mental health (3, 8), most studies have primarily focused on cognitive reappraisal and emotional well-being in relatively healthy or institutionalized older populations. There remains a limited understanding of how these emotion regulation processes interact specifically with perceived burdensomeness among community-dwelling older adults, particularly those managing chronic illness or psychosocial stressors.

Emerging evidence suggests that perceived burdensomeness is a critical psychological factor linked to reduced well-being and increased risk of mental health issues, including depression and suicidality (18, 19). While social support has been identified as a protective factor that mediates these effects (19), the role of emotion regulation as a buffer or moderating mechanism in this relationship remains underexplored. Additionally, the role of generativity as a potential mitigating factor (20) underscores the complex, multifaceted interplay of psychosocial resources that may help older adults cope with feelings of burdensomeness and emotional distress.

Thus, there is a need for comprehensive, community-based research that investigates the dynamic relationships between emotion regulation, perceived burdensomeness, and mental health in older adults. Such research is vital for developing targeted psychological and community nursing interventions aimed at improving the quality of life and emotional resilience in aging populations living independently.

### 2.1 Objectives of the study

This study aimed to examine the relationship between perceived burdensomeness, emotional regulation, and mental health among community-dwelling older adults. Specifically, it sought to assess how emotional regulation mediates the impact of perceived burdensomeness on mental health outcomes.

### 2.2 Research questions

1. What is the relationship between perceived burdensomeness and emotional regulation in older adults?
2. How does perceived burdensomeness relate to mental health outcomes among older adults?
3. Does emotional regulation mediate the relationship between perceived burdensomeness and mental health?

## 3 Methods

### 3.1 Study design and setting

The study employed cross-sectional descriptive research design and followed the Strengthening the Reporting of Observational Studies in Epidemiology (STROBE) guidelines to ensure methodological rigor and transparency. It was carried out at the outpatient clinics of Zagazig University Hospitals, located in Sharkia Governorate, Egypt. These hospitals are a prominent tertiary care center in the region, serving a diverse population from both urban and rural areas of Sharkia and surrounding governorates. Known for providing a wide range of specialized medical services, Zagazig University Hospitals play a vital role in delivering comprehensive healthcare in the region. The outpatient clinics, in particular, are well-equipped to manage a variety of medical conditions, including those commonly seen in older adults.

### 3.2 Sample size and study population

The inclusion criteria for this study required participants to be community-dwelling older adults aged 60 years and above, residing in Sharkia Governorate, Egypt, and capable of providing informed consent. Eligible individuals had to be mentally competent, able to understand and respond to interview questions, and willing to participate voluntarily. The exclusion criteria excluded individuals residing in nursing homes or long-term care facilities, those with severe cognitive impairments, as assessed by a brief cognitive screening tool, and those unable to communicate effectively due to language or significant sensory impairments. Participants diagnosed with severe psychiatric disorders, such as schizophrenia or bipolar disorder, or those who were undergoing psychiatric treatment were also excluded to avoid confounding influences on emotional regulation and mental health outcomes.

**Figure 1:**
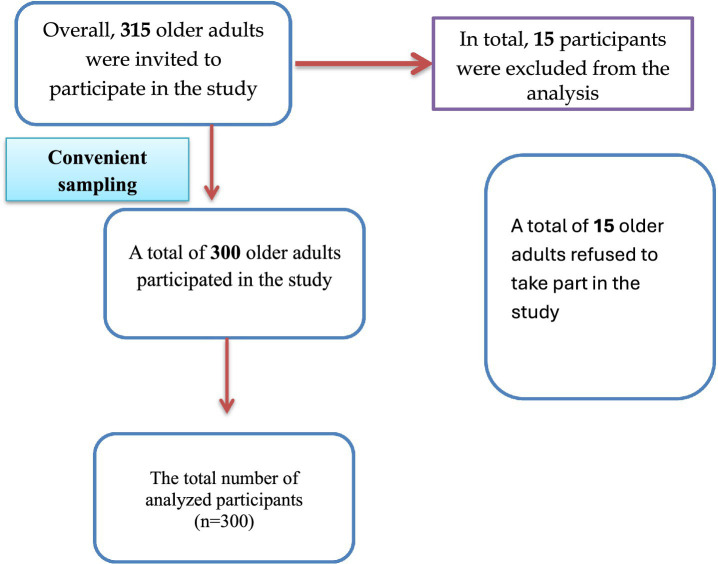
Participant’s recruitment flowchart.

The required sample size was determined *a priori* using G*Power 3.1 software (21) for multiple regression analysis, assuming a medium effect size (*f*^2^ = 0.15), *α* = 0.05, and statistical power (1 − *β*) = 0.84. The analysis indicated that at least 300 participants were required. To compensate for potential nonresponse, 315 individuals were recruited. Fifteen eligible individuals declined participation, resulting in a final analytic sample of 300 participants (Figure 1).

### 3.3 Instruments

#### 3.3.1 The demographic form

This form encompasses various aspects, including socio-demographic details such as age, gender, education level, marital status, living arrangements, family structure, employment status, and income. It also delves into medical history, covering chronic health conditions and the duration of diagnoses. Furthermore, lifestyle factors like exercise routines and risk behaviors, such as smoking, are evaluated.

#### 3.3.2 The geriatric feelings of burdensomeness scale (GFBS)

The Geriatric Feelings of Burdensomeness Scale (GFBS), created by Lutz et al. (22), is designed to assess feelings of burdensomeness in older adults. It consists of 25 items rated on a five-point Likert scale, ranging from strongly disagree (1) to strongly agree (5). The total score can range from 25 to 125, with higher scores indicating stronger feelings of burdensomeness. Examples of items from the scale; It is financially difficult for my loved ones to care for me and I am a burden on others.

According to Lutz et al. (22), the scale has shown excellent internal consistency, with a Cronbach’s alpha of 0.97. Its construct validity was confirmed through both exploratory and confirmatory factor analyses, validating the GFBS as a reliable and accurate tool for measuring burdensomeness in older adults. In this study, the GFBS demonstrated strong reliability with a Cronbach’s alpha of 0.87.

#### 3.3.3 The emotion regulation questionnaire (ERQ)

The Emotion Regulation Questionnaire (ERQ) is a self-report instrument with 10 items designed to evaluate individuals’ tendencies to use two distinct emotion regulation strategies: Cognitive Reappraisal and Expressive Suppression. Cognitive Reappraisal involves changing one’s perspective on a situation to regulate emotions (e.g., “I control my emotions by altering the way I think about the situation I’m in”), while Expressive Suppression refers to restraining the outward display of emotions (e.g., “I control my emotions by not expressing them”). Participants rate their agreement with each statement on a 7-point Likert scale, ranging from 1 (strongly disagree) to 7 (strongly agree), with higher scores reflecting greater use of the respective strategy. For this study, the Italian version of the ERQ, validated by Balzarotti, John, and Gross (23) was used. According to Balzarotti, John, and Gross (23), Cronbach’s *α* reliability coefficients were 0.84 for the Reappraisal scale and 0.72 for the Suppression scale.

In this study, the ERQ demonstrated high internal consistency, with a Cronbach’s alpha coefficient of 0.86.

#### 3.3.4 Depression anxiety stress Scale-21 (DASS-21)

The Arabic version of the Depression Anxiety Stress Scale-21 (DASS-21), translated by Ali and Green (24) for Egyptian clients, is designed to assess the distinct components of depression, anxiety, and stress experienced in the past week. This version retains the original structure of the DASS-21, consisting of 21 items divided into three subscales (25). Examples of items in the scale; I was aware of dryness of my mouth, and I felt I was close to panic. Each subscale contains seven items, which are rated on a four-point Likert scale, ranging from 0 (did not apply to me at all) to 3 (applied to me very much or most of the time). Higher scores reflect greater levels of psychological distress. The original DASS demonstrated excellent internal consistency, with Cronbach’s alpha coefficients of 0.91 for Depression, 0.84 for Anxiety, and 0.90 for Stress (25). In the present study, the Arabic version of the DASS-21 demonstrated excellent internal consistency, with a Cronbach’s alpha of 0.88.

### 3.4 Study procedures

#### 3.4.1 Tool preparation and pilot study

The research instruments, including the GFBS, ERQ, and DASS-21, were carefully translated into Arabic with a focus on accuracy and cultural relevance. Bilingual experts proficient in both English and Arabic carried out the translation to ensure precision and appropriateness. To validate the translations, a back-translation process was employed to ensure linguistic equivalence and resolve any inconsistencies. After the translation and back-translation, face validity evaluations were conducted for each instrument. Expert panels reviewed the translated tools to verify that they accurately represented the intended constructs in the Arabic context. In addition, feedback was collected from potential participants to assess the clarity, relevance, and cultural suitability of the translated items. Reliability was measured using statistical methods such as Cronbach’s alpha to confirm internal consistency. A pilot study with 30 older adults was conducted to test the clarity, relevance, and reliability of the instruments. The participants of this pilot study were excluded from the main study. The pilot study results showed no need for modifications, confirming the instruments’ appropriateness for the main research.

#### 3.4.2 Data collection

The data collection process commenced with a comprehensive orientation session for all participants, during which the study’s purpose was clearly outlined and the voluntary nature of participation was emphasized. Researchers were present to answer any questions and to reassure participants about the confidentiality protocols in place to foster a sense of trust. Data collection was carried out by trained researchers between January and March 2025. Written informed consent was obtained from each participant prior to their involvement. To maintain anonymity, participants were instructed to avoid including any personal identifiers on the questionnaires. Participation was entirely voluntary, and individuals had the right to withdraw at any stage. Data were collected through face-to-face interviews conducted in the waiting areas of clinics. Each interview lasted approximately 20 to 30 min and was scheduled between 10:00 a.m. and 2:00 p.m., from Saturday to Thursday, to accommodate participants’ schedules and ensure smooth data collection.

#### 3.4.3 Ethical considerations

The study received approval from the Research Ethics Committee of the Faculty of Nursing, Zagazig University, Egypt (code 0244). Additionally, permission was obtained from the directors of the participating clinics after providing a thorough explanation of the study’s objectives. The research was conducted in full adherence to the ethical standards outlined in the Declaration of Helsinki, ensuring the protection of participants’ rights and well-being. Before participation, all individuals were given a detailed explanation of the study’s purpose and provided written informed consent. Strict measures were implemented to safeguard participants’ privacy and ensure anonymity, with all collected data treated with the highest level of confidentiality. Participants were also informed of their right to withdraw from the study at any time without facing any negative consequences.

#### 3.4.4 Data analysis

SPSS 26.0 (IBM Inc., Chicago, IL, USA) software program was used for data analysis in order to assess the 300 participants’ answers. Descriptive statistics were used to characterize the overall characteristics of the participants and the scores on different measures. Categorical variables were shown in number and percentage and numerical variables were shown in mean ± standard deviations. To evaluate correlations between research variables, Pearson’s correlation analysis is used. Regression analysis test used to determine effect of emotional regulation as independent variable on transforms mental health and perceived burdensomeness as dependent variable in community dwelling older adults.

## 4 Results

Table 1 shows that, 39.7% of the participants had age from 65 to less than 70, 57% of them were male, 54.3% were from rural area, 68.7% were married and 44.7% had university and above. Also, 71.3% had enough income, 73.7% did not have current work, 82.7% did not smoke, 86.7% did not had regular exercise, 81.3% had chronic diseases, and 46% had chronic diseases from 5 years and more.

**Table 1:** Personal data among participants (*n* = 300).

Personal data	*N* (%)	Geriatric feelings of burdensomeness		Emotion regulation		Depression anxiety stress	
		Mean ± SD	*F*\*t*(*p*)	Mean ± SD	*F*\*t*(*p*)	Mean ± SD	*F*\*t*(*p*)
Age							
From 60 to less than 65	98 (32.7)	51.02 ± 15.20	43.148 0.000	48.21 ± 8.45	26.333 0.000	17.12 ± 10.37	46.674 0.000
From 65 to less than 70	119 (39.7)	60.89 ± 19.70		40.51 ± 12.16		22.52 ± 13.74	
70 and above	83 (27.7)	77.08 ± 21.49		35.80 ± 14.07		37.28 ± 18.73	
Gender							
Male	171 (57.0)	60.18 ± 21.48	1.842 0.067	43.18 ± 12.49	2.309 0.022	23.79 ± 17.08	1.276 0.203
Female	129 (43.0)	64.75 ± 21.09		39.79 ± 12.64		26.24 ± 15.51	
Residence							
Urban	137 (45.7)	61.66 ± 19.18	−0.360 0.719	41.59 ± 12.47	0.161 0.873	26.08 ± 15.53	1.199 0.231
Rural	163 (54.3)	62.55 ± 23.15		41.83 ± 12.83		23.80 ± 17.14	
Marital status							
Married	206 (68.7)	60.09 ± 19.73	3.260 0.040	43.41 ± 11.04	9.594 0.000	22.77 ± 15.68	5.522 0.004
Divorced	11 (3.7)	63.09 ± 26.04		47.09 ± 13.84		26.81 ± 12.97	
Widow	83 (27.7)	67.13 ± 24.03		36.81 ± 14.82		29.72 ± 17.74	
Level of education							
Illiterate	79 (26.3)	76.27 ± 21.53	18.644 0.000	36.39 ± 13.78	7.747 0.000	36.31 ± 16.54	21.245 0.000
basic education	38 (12.7)	59.73 ± 18.14		45.44 ± 9.37		19.60 ± 12.43	
Secondary	49 (16.3)	57.34 ± 21.06		41.20 ± 12.63		19.44 ± 13.95	
University and above	134 (44.7)	56.26 ± 18.50		44.00 ± 11.84		21.54 ± 15.04	
Income							
Enough	214 (71.3)	58.85 ± 19.06	31.036 0.000	42.68 ± 11.71	13.378 0.000	22.28 ± 0.15.10	14.157 0.000
Not enough	54 (18.0)	80.70 ± 20.97		34.59 ± 13.49		35.05 ± 17.29	
Enough and Save	32 (10.7)	52.84 ± 20.12		47.34 ± 12.71		24.78 ± 17.38	
Job							
Work	79 (26.3)	59.20 ± 18.06	1.429 0.154	44.02 ± 11.71	1.890 0.060	22.58 ± 15.76	1.428 0.154
not work	221 (73.7)	63.20 ± 22.41		40.90 ± 12.89		25.65 ± 16.64	
Cigarette smoking							
Yes	52 (17.3)	78.25 ± 21.65	6.346 0.000	33.69 ± 14.15	5.256 0.000	40.13 ± 18.73	8.138 0.000
No	248 (82.7)	58.77 ± 19.79		43.41 ± 11.65		21.64 ± 13.97	
Regular exercise							
Yes	40 (13.3)	46.97 ± 16.35	5.007 0.000	46.72 ± 10.54	2.712 0.007	11.25 ± 10.40	5.929 0.000
No	260 (86.7)	64.48 ± 21.15		40.95 ± 12.79		26.93 ± 16.21	
History of chronic disease							
Yes	244 (81.3)	64.66 ± 21.12	4.373 0.000	40.94 ± 12.88	2.256 0.025	27.31 ± 16.19	5.708 0.000
No	56 (18.7)	51.19 ± 19.18		45.14 ± 11.03		14.08 ± 12.91	
Duration since diagnosis with a chronic disease							
No	51 (17.0)	46.92 ± 15.23	18.256 0.000	46.52 ± 9.73	7.510 0.001	11.29 ± 9.83	24.537 0.000
less than 5 years	111 (37.0)	67.19 ± 18.76		42.84 ± 10.95		26.67 ± 14.41	
5 years and more	138 (46.0)	63.71 ± 22.86		39.05 ± 14.19		28.38 ± 17.44	

*F*, ANOVA test, *t*, independent *t*-test.

The findings demonstrated statistically significant differences in geriatric feelings of burdensomeness, emotion regulation, and depression–anxiety–stress across several sociodemographic and health-related variables. Age showed a highly significant association with all three outcomes (*p* < 0.001), with participants aged ≥70 years reporting the highest burdensomeness and depression–anxiety–stress scores and the lowest emotion regulation levels compared with younger age groups. Gender differences were significant only for emotion regulation (*p* = 0.022), where males exhibited higher emotion regulation than females. Marital status was significantly related to all outcomes (*p* < 0.05). Educational level demonstrated strong significant differences across all variables (*p* < 0.001); illiterate participants had the highest burdensomeness and depression–anxiety–stress and the poorest emotion regulation.

Income adequacy was also strongly associated with all outcomes (*p* < 0.001). Older adults reporting insufficient income had markedly higher burdensomeness and depression–anxiety–stress and lower emotion regulation compared with those having adequate or saved income. Smokers reported significantly higher burdensomeness and depression–anxiety–stress and poorer emotion regulation than non-smokers (*p* < 0.001). Similarly, participants who did not engage in regular exercise had worse psychological outcomes than those who exercised regularly (*p* < 0.01). Older adults with chronic diseases exhibited higher burdensomeness and distress and lower emotion regulation than those without chronic illness and also the history of chronic disease. Moreover, duration of chronic disease was significantly associated with all outcomes (*p* ≤ 0.001), with longer disease duration linked to greater psychological burden and poorer emotional regulation.

Table 2 shows that the mean score of the participants’ geriatric feelings of burdensomeness scale is 62.15 ± 21.40 and its subscale cognitive reappraisal was 25.64 ± 7.98 and expressive suppression was 16.08 ± 5.62. Also, emotion regulation was 41.72 ± 12.65. Furthermore, the mean score of the participant depression, anxiety and stress was 24.84 ± 16.44. Also, the mean score of the participants’ Anxiety was 7.67 ± 5.68, Stress was 9.02 ± 5.92 and Depression was 8.15 ± 5.92.

**Table 2:** Descriptive analysis between study variables (*n* = 300).

Variables	*N*	Minimum	Maximum	Mean	Std. deviation	*Α*
Geriatric Feelings of Burdensomeness	25	25.00	125.00	62.15	21.40	0.782
Emotion Regulation	10	10.00	67.00	41.72	12.65	0.894
Cognitive Reappraisal	6	6.00	41.00	25.64	7.98	0.785
Expressive Suppression	4	4.00	28.00	16.08	5.62	0.853
Depression Anxiety Stress	21	0.00	63.00	24.84	16.44	0.939
Anxiety	7	0.00	21.00	7.67	5.68	0.850
Stress	7	0.00	21.00	9.02	5.92	0.923
Depression	7	0.00	21.00	8.15	5.92	0.893

*α* = Cronbach’s alpha, SD = Std. deviation.

Table 3 clarifies that there was a statistically significant positive correlation between the participant geriatric feelings of burdensomeness scale and depression anxiety stress (*r* = 0.765^**^, *p* < 0.001), while that, there was a statistically significant negative correlation of the participant geriatric feelings of burdensomeness and emotion regulation scale (*r* = −0.676^**^, *p* < 0.001), and between emotion regulation and depression anxiety stress (*r* = −0.614^**^, *p* < 0.001).

**Table 3:** Correlation between study variables among participants (*n* = 300).

Variables		1	2	3
1. Geriatric feelings of burdensomeness	*r*	1		
	*p*			
2. Emotion regulation	*r*	−0.676-**	1	
	*p*	<0.001		
3. Depression anxiety stress	*r*	0.765^**^	−0.614-**	1
	*p*	<0.001	<0.001	

*r* = Pearson correlation, ** correlation is highly significant at the 0.01 level (2-tailed).

Table 4 shows the factors that play as predictors for participants’ burdensomeness and depression, anxiety and stress, regarding model 1 “burdensomeness” the study finding indicated that emotion regulation at (*B* = −0.91, Beta = −0.54, *t* = −12.20, *p* < 0.001) play as predictors for participants’ burdensomeness, Furthermore, age at (*B* = 4.70, Beta = 0.170, *t* = 3.73, *p* < 0.001), educational level at (*B* = −2.60, Beta = −0.153, *t* = −3.41, *p* = 0.001), regular exercise at (*B* = −5.95, Beta = 0.095, −*t* = 2.32, *p* = 0.021), history of chronic disease at (*B* = 0.11.23, Beta = 0.205, *t* = −3.50, *p* = 0.001) play as predictors for participants’ burdensomeness, also, the model summary values were *R* = 0.763, *R* Square = 0.583, *F* = 33.371 at *p* < 0.00.

**Table 4:** Regression model of the effect of emotional regulation on the participants burdensomeness and depression, anxiety and stress (*n* = 300).

Variables	Model 1 “Burdensomeness”							Model 2 “Depression, anxiety and stress”						
	*B*	Std. Error	Beta	*t*	*p*	95.0%CI		*B*	Std. error	Beta	*t*	*p*	95.0%CI	
						Lower bound	Upper bound						Lower bound	Upper bound
(Constant)	114.4	12.04		9.50	**<0.001**	90.76	138.19	53.91	9.43		5.71	**0.000**	35.34	72.48
Age	4.70	1.25	0.170	3.73	**<0.001**	2.22	7.17	2.60	0.98	0.123	2.64	**0.009**	0.668	4.55
Gender	2.25	2.16	0.052	1.04	0.297	−2.00	6.51	0.53	1.69	0.016	0.31	0.752	−2.79	3.87
Residence	1.69	1.77	0.040	0.95	0.339	−1.79	5.18	−1.10	1.38	−0.03	−0.79	0.426	−3.83	1.62
Marital status	−0.314	1.06	−0.013	−0.29	0.769	−2.41	1.79	0.892	0.83	0.048	1.06	0.287	−0.75	2.54
Level of education	−2.60	0.76	−0.153	−3.41	**0.001**	−4.10	−1.10	−1.15	0.59	−0.088	−1.93	0.055	−2.33	0.023
Income	0.448	1.31	0.014	0.341	0.733	−2.13	3.03	−2.66	1.02	0.109	−2.59	**0.010**	0.642	4.68
Job	−0.291	2.07	−0.006	−0.140	0.889	−4.37	3.79	−1.46	1.62	−0.039	−0.90	0.369	−4.65	1.73
Cigarette Smoking	−5.03	2.64	−0.089	−1.90	0.058	−10.24	0.17	8.22	2.07	0.19	3.96	**<0.001**	−12.30	−4.14
Regular exercise	−5.95	2.56	−0.095	−2.32	**0.021**	0.908	11.00	−6.51	2.00	−0.135	−3.24	**0.001**	2.56	10.46
History of chronic disease	11.23	3.20	0.205	3.50	**0.001**	−17.54	−4.93	7.02	2.50	−0.167	2.80	**0.005**	−11.96	−2.08
Duration since diagnosis with a chronic disease	−4.29	1.75	−0.148	−2.44	**0.015**	−7.75	−0.83	0.56	1.37	0.025	0.409	0.683	−2.14	3.27
Emotion Regulation	−0.91	0.07	−0.54	−12.20	**<0.001**	−1.06	−0.76	−0.56	0.05	−0.434	−9.63	**<0.001**	−0.68	−0.449

	Summary model	Summary model
*R*	0.763	0.753
*R* square	0.583	0.567
*F* (*p*)	33.371 (**<0.001**)	31.259 (**<0.001**)

*B*, unstandardized coefficients; Beta, standardized coefficients; CI, confidence interval.

Bold values indicate statistical significance at *p* < 0.05.

Concerning Model 2”Depression, Anxiety and Stress” the study finding indicated that emotion regulation at (*B* = −0.56, Beta = −0.434, *t* = −9.63, *p* < 0.001) have direct signifies effect on participants depression, anxiety and stress. Furthermore, age at (*B* = 2.60, Beta = 0.123, *t* = 2.64, *p* = 0.009), income at (*B* = 2.66, Beta = 0.109, *t* = 2.59, *p* = 0.010), cigarette smoking at (*B* = 8.22, Beta = −0.19, *t* = −3.96, *p* < 0.001), regular exercise at (*B* = −6.51, Beta = 0.135, *t* = −3.24, *p* = 0.001), history of chronic disease at (*B* = 0.02, Beta = 0.167, *t* = 2.80, *p* = 0.005) had direct signifies effect on participants depression, anxiety and stress, also, the model summary values were *R* = 0.753, *R* Square = 0.567, *F* = 31.259 at *p* < 0.00.1.

## 5 Discussion

This study aimed to investigate the relationship between perceived burdensomeness, emotional regulation, and mental health among community-dwelling older adults. The findings underscored the complex interplay between these variables, revealing that higher levels of burdensomeness were strongly associated with increased emotional dysregulation and poorer mental health outcomes. Conversely, emotional regulation—particularly adaptive strategies like cognitive reappraisal—acted as a protective factor, mediating the negative impact of burdensomeness on mental well-being. These insights contribute significantly to geriatric psychology by highlighting the dual role of burdensomeness as both a symptom and a predictor of psychological distress and suggesting emotion regulation as a potential therapeutic target.

The relationship between perceived burdensomeness and emotional regulation emerged as a core finding, revealing that older adults who view themselves as a burden exhibit significantly poorer emotional regulation capabilities. This connection may stem from the internalization of negative self-perceptions—such as feeling useless or unwanted—which fosters chronic emotional arousal and impairs the ability to process or regulate affect (26). Participants who endorsed higher levels of burdensomeness struggled more with emotional clarity, impulse control, and acceptance of emotional responses (27).

While aging theories, such as Socioemotional Selectivity Theory (6), posit that emotional regulation improves with age, our findings challenge this assumption. We observed that burdensomeness can disrupt expected emotional gains, echoing recent work by Gager et al. (20), who demonstrated that burdensomeness in older adults was linked to reduced generativity and increased emotional vulnerability. This suggests that interpersonal perceptions can counteract otherwise normative emotional growth in older age.

These findings also question the dominant framing of emotional dysregulation as a fixed trait. Instead, our results portray it as context-sensitive, particularly vulnerable to social-cognitive stressors like burdensomeness (19). In contrast to Starkey et al. (28), who explored burdensomeness primarily in younger populations and found emotion regulation to be more trait-dependent, our data point to a reactive model in older adults. The emotional fragility observed here under burdensome perceptions underscores the need to address root cognitive beliefs—not merely surface-level symptoms (29). Interventions must thus extend beyond skills training to actively challenge the internalized notion of being a burden, which appears to erode emotional resilience even in older adults.

This study further highlights emotional regulation as a pivotal predictor of mental health among older adults, affirming that those with better regulation skills report significantly lower levels of psychological distress. Specifically, emotional clarity, impulse control, and goal-directed behavior under emotional strain emerged as key buffers against depression and anxiety. These findings align with Gross (3) and Naragon-Gainey et al. (36), reinforcing that emotional competence fosters psychological resilience. However, our results deepen this understanding by showing that in late life—when social roles narrow and existential concerns rise—emotion regulation becomes even more vital. Notably, our findings mirror those of Depoorter et al. (30), who found that poor regulation strategies, such as rumination and suppression, are uniquely predictive of depressive symptoms in older adults. This convergence suggests that emotion regulation may be particularly consequential in later life stages, contradicting assumptions that its influence wanes with age.

While younger individuals often have access to external resources for stress buffering, such as employment or peer networks, older adults are increasingly reliant on internal mechanisms like emotion regulation (31). Contrary to theories that suggest emotional stability plateaus or improves with age, we found that these skills remain plastic and crucial. This aligns with Amin et al. (19), who found that social support enhances the positive effect of emotion regulation on well-being in older adults. Our study adds to this by suggesting that regulation strategies should be dynamically reinforced, not presumed intact, especially when cognitive-emotional threats like burdensomeness are present. Clinicians and caregivers must therefore view emotional regulation as a modifiable, lifelong skill with significant implications for psychological functioning in later years.

The data also confirm a strong positive relationship between perceived burdensomeness and poor mental health outcomes. Participants who felt that they were liabilities to their families or communities exhibited heightened symptoms of depression, anxiety, and general emotional distress. This result echoes Van Orden et al.’s (12) Interpersonal Theory of Suicide, which identifies perceived burdensomeness as a potent driver of suicidal ideation. Yet, our study broadens this model by demonstrating that burdensomeness also predicts general psychological distress in non-clinical, community-dwelling older adults. Supporting this, Li et al. (32) found that negative self-perceptions around aging—including burdensomeness—were significantly associated with emotional distress among older Chinese adults, highlighting the global relevance of these cognitive-emotional constructs. Unlike physical illness or bereavement, burdensomeness operates at a psychological level and is thus potentially amenable to cognitive restructuring (33).

While traditional models of geriatric mental health have focused on physical deterioration, grief, or loss of independence, our findings bring attention to the deeply relational and modifiable belief of being a burden. Gager et al. (20) further support this relational framing, finding that generativity—feeling useful and contributing—buffers the harmful impact of perceived burdensomeness. Our findings compel a shift from managing symptoms toward affirming social value and agency in older adults. Interventions targeting identity reconstruction and role affirmation may be more effective in reducing distress than those limited to symptom relief. By centering the psychosocial dimension of burdensomeness, we introduce a critical pivot in how mental health services are conceptualized for aging populations.

Another major insight from this study is that emotional regulation significantly mediated the relationship between perceived burdensomeness and mental health outcomes. Participants with stronger regulation capacities reported fewer symptoms of depression and anxiety—even when they experienced moderate burdensomeness. This finding aligns with the Socioemotional Selectivity Theory, which emphasizes the prioritization of emotionally meaningful experiences in older adulthood (34). Emotional clarity and impulse control specifically emerged as crucial mediators, consistent with Gross’s (3) argument that adaptive regulation strategies—like reappraisal—diminish the duration and intensity of negative emotions. Zhu et al. (35) corroborate this mechanism, suggesting that older adults who engage in adaptive emotion regulation are better equipped to cope with relational stressors.

What sets our study apart is its identification of emotion regulation as a dynamic buffer—not a static trait—within the burdensomeness-mental health pathway. This adds nuance to previous work, such as that of Carstensen (6), by showing that emotional wisdom can be compromised by chronic negative self-appraisals. Even emotionally adept individuals may falter under sustained burdensomeness, highlighting the limitations of internal resources when social validation is lacking. This interaction underscores the necessity for integrative interventions that simultaneously bolster regulation skills and reframe burdensome beliefs. Our findings suggest that promoting emotion regulation alone may be insufficient without addressing the cognitive-emotional roots of burdensomeness. By illuminating this dual target—skill-building and belief transformation—we offer a more comprehensive roadmap for enhancing psychological resilience in older adults.

Demographic and health-related characteristics also played a significant role in shaping psychological outcomes. Advanced age was associated with higher perceived burdensomeness and greater psychological distress, alongside lower emotion regulation capacity. This may reflect increased functional limitations, health decline, and social dependency in later stages of aging. Gender differences were observed in emotion regulation, with males reporting higher regulation abilities than females. Although psychological distress did not significantly differ by gender, women demonstrated slightly higher distress scores, which may be influenced by longer life expectancy, widowhood, and caregiving burdens. Marital status was another influential factor, as widowed participants exhibited poorer psychological outcomes compared to married individuals, likely due to reduced emotional support and increased loneliness. Furthermore, the presence of chronic disease significantly predicted both burdensomeness and psychological distress, highlighting the interconnected relationship between physical health and emotional well-being in older adulthood.

By identifying perceived burdensomeness and emotion regulation as modifiable risk factors that can be addressed at the population level among older adults, these findings go beyond individual or clinical care from a public health perspective. It may be possible to lessen psychological distress, enhance emotional well-being, and lower the risk of suicide in older populations by incorporating emotion regulation training into community-based programs, primary care settings, and public health initiatives. Public health initiatives should also concentrate on lowering social isolation, bolstering social support, and encouraging active aging because these elements have a direct impact on mental health outcomes and perceived burdensomeness.

### 5.1 Implications of the study

The findings of this study have important implications from a public health perspective, particularly in the context of aging populations and the growing burden of mental health conditions among older adults. The results underscore the need for population-level strategies that address emotional regulation as a key determinant of mental well-being. Integrating emotional regulation interventions into community-based mental health programs, primary healthcare services, and aging support systems may help reduce psychological distress and perceived burdensomeness among older adults. Such approaches align with preventive public health models that emphasize early identification, health promotion, and risk reduction.

At the community level, public health initiatives should focus on strengthening social support, reducing stigma associated with mental health, and promoting active and healthy aging. Programs that incorporate psychoeducation, lifestyle modification (e.g., physical activity and smoking cessation), and emotional resilience training can contribute to improved mental health outcomes and enhanced quality of life among older adults. Additionally, routine mental health screening within primary care and community settings can facilitate early detection and timely intervention, particularly for vulnerable groups such as those with chronic illnesses or limited social support. From a health systems perspective, interdisciplinary collaboration among healthcare providers—including nurses, physicians, psychologists, and public health professionals—is essential to deliver comprehensive and person-centered care. While nursing plays a critical role in implementation, broader system-level integration is required to ensure sustainability and scalability of interventions.

Furthermore, these findings highlight the need for future research to evaluate the effectiveness of community-based and population-level interventions targeting emotional regulation and perceived burdensomeness. Exploring digital health solutions, public awareness campaigns, and policy-driven approaches may further support mental health promotion in aging societies. Overall, addressing emotional regulation and perceived burdensomeness through a public health lens can contribute to reducing the burden of depression and anxiety, preventing suicide risk, and promoting healthier and more resilient aging populations.

### 5.2 Strengths and limitations

This study demonstrates several strengths, particularly in the use of psychometrically sound and validated instruments. The Geriatric Feelings of Burdensomeness Scale (GFBS), Emotion Regulation Questionnaire (ERQ), and Depression Anxiety Stress Scale-21 (DASS-21) have established reliability and validity, making them appropriate tools for measuring the key variables in this study. Additionally, the instruments were carefully translated and culturally adapted for the Arabic-speaking population, ensuring relevance and accuracy in assessing emotional regulation, mental health, and burdensomeness. The study also includes a pilot testing phase, which confirmed the clarity, relevance, and reliability of the instruments before their application to the larger sample. Furthermore, the study adhered to strict ethical standards, with informed consent and confidentiality protocols in place to protect participants’ rights. The study’s sample size, determined through power analysis, was sufficient to detect medium effect sizes, thereby enhancing the robustness of the results.

Despite these strengths, the study has some limitations. The cross-sectional design limits the ability to infer causal relationships between emotional regulation, perceived burdensomeness, and mental health outcomes, as it captures data at a single point in time. Additionally, the reliance on self-report instruments introduces the potential for social desirability bias or inaccuracies, as participants may not fully disclose sensitive information regarding their mental health or emotional regulation. The exclusion criteria further restrict the generalizability of the findings, as individuals with cognitive impairments or severe psychiatric conditions were excluded, which may overlook important aspects of the older adults. Lastly, although the study was conducted at a prominent tertiary care center in Sharkia Governorate, the findings may not be fully applicable to other regions or populations with different socio-cultural contexts, limiting the broader applicability of the results.

## 6 Conclusion

This study highlights that emotion regulation is a key determinant of mental health among older adults, with important implications for public health improvement and policy. From a population health perspective, the findings emphasize the need to integrate mental health promotion and emotional regulation strategies into community-based and primary healthcare services targeting aging populations. Public health policies should prioritize early screening for psychological distress, strengthen social support systems, and expand access to preventive, community-level interventions that reduce perceived burdensomeness and enhance resilience. In addition, addressing broader social determinants such as chronic illness, income insecurity, and social isolation is essential for improving mental well-being in later life. Embedding these approaches within national healthy aging strategies can contribute to reducing the burden of depression and anxiety, preventing suicide risk, and promoting healthier, more inclusive aging societies.

## Data Availability

The raw data supporting the conclusions of this article will be made available by the authors, without undue reservation.

## References

[1] Institute of Health Metrics and Evaluation. (2023). Global Health data exchange (GHDx). Available online at: https://vizhub.healthdata.org/gbd-results/

[2] Hong Teo R Hui Cheng W Jie Cheng L Lau Y Tiang Lau S. Global prevalence of social isolation among community-dwelling older adults: a systematic review and meta-analysis. Arch Gerontol Geriatr. (2023) 107:104904. doi: 10.1016/j.archger.2022.104904, 36563614

[3] Gross JJ. Emotion regulation: current status and prospects. Psychol Inq. (2015) 26:1–26. doi: 10.1080/1047840X.2014.940781

[4] Allen V Windsor T. Age differences in the use of emotion regulation strategies derived from the process model of emotion regulation: a systematic review. Aging Ment Health. (2019) 23:1–14. doi: 10.1080/13607863.2017.1396575, 29148830

[5] Riediger M Bellingtier JA. Emotion Regulation Across the Lifespan. Germany: Department of Developmental Psychology, University of Jena (2020).

[6] Carstensen LL. The influence of a sense of time on human development. Science. (2006) 312:1913–5. doi: 10.1126/science.1127488, 16809530 PMC2790864

[7] Urry HL Gross JJ. Emotion regulation in older age. Curr Dir Psychol Sci. (2010) 19:352–7. doi: 10.1177/0963721410388395

[8] Christopher G Facal D. Editorial: emotion regulation and mental health in older adults. Front Psychol. (2023) 14:1173314. doi: 10.3389/fpsyg.2023.1173314, 37034944 PMC10080131

[9] Drapeau CW McIntosh JL. USA Suicide 2013: Official Final Data. Washington, DC: American Association of Suicidology (2015).

[10] Friedmann H Kohn R. Mortality, or probability of death, from a suicidal act in the United States. Suicide Life Threat Behav. (2008) 38:287–301. doi: 10.1521/suli.2008.38.3.287, 18611127

[11] Joiner T. Why People Die by Suicide. Cambridge, MA: Harvard University Press (2005).

[12] Van Orden KA Witte TK Cukrowicz KC Braithwaite SR Selby EA Joiner TE. The interpersonal theory of suicide. Psychol Rev. (2010) 117:575–600. doi: 10.1037/a0018697, 20438238 PMC3130348

[13] Van Orden KA Lynam ME Hollar D Joiner TE. Perceived burdensomeness as an indicator of suicidal symptoms. Cogn Ther Res. (2006) 30:457–67. doi: 10.1007/s10608-006-9057-2

[14] Van Orden KA Witte TK Gordon KH Bender TW Joiner TE. Suicidal desire and the capability for suicide: tests of the interpersonal-psychological theory of in perceived burdensomeness in young and old adults (2008) 76:72–83. doi: 10.1037/0022-006X.76.1.72, 18229985

[15] Asal MGR El-Sayed AAI Alsenany SA Ramzy ZH Dawood RFA. Self-administered active versus sham acupressure for diarrhea predominant irritable bowel syndrome: a nurse-led randomized clinical trial. BMC Nurs. (2025) 24:106. doi: 10.1186/s12912-024-02594-5, 39875940 PMC11776273

[16] Christensen H Batterham PJ Soubelet A Mackinnon AJ. A test of the interpersonal theory of suicide in a large community-based cohort. J Affect Disord. (2013) 144:225–34. doi: 10.1016/j.jad.2012.07.002, 22862889

[17] Hill RM Pettit JW. Perceived burdensomeness and suicide-related behaviors in clinical samples: current evidence and future directions. J Clin Psychol. (2014) 70:631–43. doi: 10.1002/jclp.22071, 24421035

[18] Jahn DR Van Orden KA Cukrowicz KC. Perceived burdensomeness in older adults and perceptions of burden on spouses and children. Clin Gerontol. (2013) 36:451–9. doi: 10.1080/07317115.2013.816817, 24179315 PMC3809949

[19] Amin SM Khedr MA Tawfik AF Noaman Malek MG El-Ashry AM. The mediating and moderating role of social support on the relationship between psychological well-being and burdensomeness among elderly with chronic illness: community nursing perspective. BMC Nurs. (2025) 24:156. doi: 10.1186/s12912-025-02743-4, 39930516 PMC11812208

[20] Gager CT Gunn JF Goldstein SE Martinez SM. Thwarted belonging and perceived burdensomeness during middle and older adulthood: the role of generativity. Int J Aging Hum Dev. (2024) 99:25–46. doi: 10.1177/00914150231208688, 38291615

[21] Faul F Erdfelder E Lang AG Buchner A. G*power 3: a flexible statistical power analysis program for the social, behavioral, and biomedical sciences. Behav Res Methods. (2007) 39:175–91. doi: 10.3758/BF03193146, 17695343

[22] Lutz J Katz E Gallegos J Spalding R Edelstein B. The geriatric feelings of burdensomeness scale (GFBS). Clin Gerontol. (2022) 45:696–707. doi: 10.1080/07317115.2020.1838982, 33245252 PMC8155102

[23] Balzarotti S John OP Gross JJ. An Italian adaptation of the emotion regulation questionnaire. Eur J Psychol Assess. (2010) 26:61–7. doi: 10.1027/1015-5759/a000009

[24] Ali AM Green J. Factor structure of the depression anxiety stress Scale-21 (DASS-21): Unidimensionality of the Arabic version among Egyptian drug users. Subst Abuse Treat Prev Policy. (2019) 14:40–8. doi: 10.1186/s13011-019-0226-1, 31533766 PMC6751677

[25] Lovibond SH. Manual for the depression anxiety stress scales.Sydney Psychology Foundation (1995).

[26] Weiss NH Dixon-Gordon KL Brick LA Goldstein SC Schick MR Laws H . Measuring emotion dysregulation in daily life: an experience sampling study. Anxiety, Stress, & Coping. (2025) 38:17–35. doi: 10.1080/10615806.2024.2366031, 38932637 PMC11671609

[27] Kaur K Lohani M Williams P Asnaani A. A person-specific emotion regulation flexibility framework: taking an integrative approach. Emot Rev. (2025) 17:17540739251323517. doi: 10.1177/17540739251323517

[28] Starkey A Bolner J Hill R. A mixed-methods examination of perceived burdensomeness in emerging adults. J Aggress Maltreat Trauma. (2025) 34:79–98. doi: 10.1080/10926771.2024.2434916

[29] Shimizu K Aung MN Moolphate S Aung TNN Koyanagi Y Supakankunti S . Substantial impact of later-life depression among community older adults on the family caregivers’ burden in the home care setting of Chiang Mai, northern Thailand. Medicina (Kaunas). (2025) 61:50. doi: 10.3390/medicina61010050, 39859032 PMC11766950

[30] Depoorter J De Raedt R Berking M Hoorelbeke K. Specificity of emotion regulation processes in depression: a network analysis. Cogn Ther Res. (2025) 49:312–23. doi: 10.1007/s10608-024-10530-9

[31] Rodrigues C Carreira S Novais R Braga F Martins S Araújo O. The mental health of older people living in nursing homes in northern Portugal: a cross-sectional study protocol. Nurs Rep. (2025) 15:24. doi: 10.3390/nursrep15010024, 39852646 PMC11767289

[32] Li J Xu W Wang Q Zhou X Peng C. Physical health problems, views on ageing, and emotional distress among older Chinese population: a moderated mediation model. Aging Ment Health. (2025) 29:747–56. doi: 10.1080/13607863.2024.2448212, 39784334

[33] Zadworna M Ardelt M. Understanding mental health in older adults: exploring the interplay of wisdom, perceived poor health, and attitudes toward aging. Aging Ment Health. (2025) 29:1485–96. doi: 10.1080/13607863.2025.2452943, 39851093

[34] Gerino E Rollè L Sechi C Brustia P. Loneliness, resilience, mental health, and quality of life in old age: a structural equation model. Front Psychol. (2017) 8:2003. doi: 10.3389/fpsyg.2017.02003, 29184526 PMC5694593

[35] Zhu K Guo Z Zhang Y Li S Wang X Xu R . Latent profile analysis of emotional inhibition in older adults with gastrointestinal tumors: a cross-sectional study. Asia Pac J Oncol Nurs. (2025) 12:100677. doi: 10.1016/j.apjon.2025.100677, 40144345 PMC11937281

[36] Naragon‐Gainey K Simms LJ. Clarifying the links of conscientiousness with internalizing and externalizing psychopathology. J. Pers. (2017) 85:880–892. doi: 10.1111/jopy.12295, 27884039 PMC5443702

